# The APOA1-SNCA Axis as a Molecular Bridge Between CKD and Parkinson’s Disease: A Systems Biology Model of Kidney-to-Brain Propagation via Exosomal Pathways

**DOI:** 10.3390/ijms27104176

**Published:** 2026-05-08

**Authors:** Deryanaz Billur, Hasmet Ayhan Hanagası, Basar Bilgic, Ozlem Timirci-Kahraman

**Affiliations:** 1 Department of Molecular Medicine, Aziz Sancar Institute of Experimental Medicine, Istanbul University, 34093 Istanbul, Türkiye; deryanazbillur@gmail.com; 2 Department of Molecular Medicine, Institute of Graduate Studies in Health Sciences, Istanbul University, 34126 Istanbul, Türkiye; 3 Department of Neurology, Faculty of Medicine, Istanbul University, 34093 Istanbul, Türkiye

**Keywords:** Parkinson’s disease, chronic kidney disease, α-synuclein, APOA1, systems biology, protein–protein interaction network, exosomes, kidney–brain axis, neuroinflammation, neurodegeneration

## Abstract

Chronic kidney disease (CKD) is an established risk factor for Parkinson’s disease (PD), but the molecular mechanisms linking these two conditions remain elusive. We performed a systems biology analysis by retrieving high-confidence gene–disease associations from DisGeNET v7.0 (PD: score ≥ 0.8, EI ≥ 0.4; CKD: score ≥ 0.6, EI ≥ 0.4) and constructing a protein–protein interaction (PPI) network via STRING v11.5 (confidence ≥ 0.700). Direct “molecular bridges” between CKD and PD proteins were identified and validated using independent databases. To corroborate biological feasibility, candidate proteins were cross-referenced with ExoCarta and Vesiclepedia databases for exosomal localization. Functional enrichment, tissue expression, and pathway analyses were conducted. Despite zero gene overlap (64 PD genes, 17 CKD genes), the PPI network showed significant convergence (81 nodes, 280 edges, PPI enrichment *p* < 1.0 × 10^−16^). Fifteen high-confidence molecular bridges were identified, including the Apolipoprotein A1 (APOA1)–α-synuclein (SNCA) interaction (combined score 0.883), which was independently validated by IntAct. Functional enrichment revealed specific association of APOA1–SNCA with “amyloid fiber formation” (false discovery rate (FDR) = 0.038). Both APOA1 and SNCA are annotated as exosome components (Kyoto Encyclopedia of Genes and Genomes (KEGG) ko04147) and were confirmed as consistent cargo in plasma, urine, and platelet-derived extracellular vesicles within proteomic databases (ExoCarta IDs: 335, 6622). Global pathway analysis highlighted inflammation, oxidative stress, and the advanced glycation end product (AGE)–receptor for AGE (RAGE) pathway. We propose an integrative model wherein CKD-induced dysregulation of APOA1 promotes α-synuclein misfolding and aggregation, and the co-packaging of these proteins into exosomes provides a plausible vehicle for kidney-to-brain propagation. This framework offers testable hypotheses and potential therapeutic targets for PD-CKD comorbidity.

## 1. Introduction

Parkinson’s disease (PD) ranks as the second most prevalent neurodegenerative condition worldwide. Its neuropathological hallmarks include the progressive degeneration of dopaminergic neurons within the substantia nigra and the accumulation of abnormal protein aggregates known as Lewy bodies, which are primarily composed of α-synuclein (SNCA) [1,2]. While PD has long been viewed as a disorder confined to the central nervous system, an expanding body of evidence indicates that its pathogenesis involves intricate cross-talk between the brain and various peripheral organs [3,4]. Chronic kidney disease (CKD) currently affects nearly 10% of adults globally and has increasingly been recognized as a substantial risk factor for PD [5,6]. Data from large-scale population-based studies reveal that individuals with CKD face a 1.5- to 2.5-fold elevated likelihood of developing PD, with the degree of risk correlating with the severity of renal impairment [7,8]. Nevertheless, the specific molecular pathways through which kidney dysfunction contributes to nigrostriatal degeneration remain largely unknown.

The notion of a “kidney–brain axis” has gained traction from clinical observations of neurological complications in patients with uremia, as well as from reports of renal dysfunction in individuals with neurodegenerative diseases [9,10]. In a pivotal recent investigation, Yuan and colleagues demonstrated that peripherally administered α-synuclein can travel from the kidney to the brain via vagal routes, thereby providing experimental support for an anatomically plausible mechanism of kidney-to-brain pathology dissemination [11]. Thus, although the clinical link between kidney disease and brain pathology is firmly established, the precise molecular substrates orchestrating this inter-organ communication have remained enigmatic—a ‘black box’ that we aim to open in the present study.

Building on this important finding, we postulated that distinct molecular substrates must exist to facilitate—and possibly enhance—the kidney-to-brain spread of α-synuclein pathology under CKD conditions. Although Yuan and co-workers convincingly showed that peripheral α-synuclein originating in the kidney can reach the central nervous system [11], the precise molecular factors that govern or accelerate this process—particularly in the setting of a chronic disease state such as CKD—have yet to be identified. Accordingly, we set out to uncover protein–protein interaction links between CKD and PD that could act as mechanistic connectors, concentrating on molecules that (i) become dysregulated during CKD, (ii) engage in direct physical association with α-synuclein, and (iii) possess the capacity to participate in inter-organ transport. Through a systems biology strategy, we aimed to evaluate the hypothesis that CKD-evoked disturbances in systemic lipid metabolism—especially those involving Apolipoprotein A1 (APOA1)—generate a permissive microenvironment that fosters α-synuclein misfolding and aggregation, thereby promoting its transmission along the kidney–brain axis.

Systems biology methodologies have proven highly effective for revealing hidden relationships among complex diseases by exploiting the organizational principles of the human interactome [12,13]. Network-oriented analyses can expose convergent molecular mechanisms emerging from the functional interplay of distinct disease-associated proteins [14,15]. Such an approach is especially well-suited for investigating comorbidities such as CKD and PD, where shared pathophysiology may stem not from overlapping genetic susceptibilities but rather from functional crosstalk between different sets of proteins.

APOA1, the primary constituent of high-density lipoprotein (HDL) particles, has recently attracted interest owing to its potential roles in both renal and neurological disorders [16,17]. Within the context of CKD, APOA1 levels and functional properties are frequently altered as a consequence of deranged lipid metabolism and persistent systemic inflammation [18]. In PD, APOA1 has been implicated in α-synuclein homeostasis, with several reports suggesting that APOA1 can physically associate with α-synuclein and modulate its propensity to aggregate [19,20]. Nonetheless, whether APOA1 might serve as a molecular bridge linking renal dysfunction to α-synuclein pathology has not been systematically explored.

In the present study, we employed a comprehensive systems biology framework to systematically map and characterize molecular connections between CKD and PD. By integrating high-confidence gene–disease associations with protein–protein interaction networks, our specific objectives were to: (1) assess whether genetically distinct disease-associated gene sets converge at the level of the protein interactome; (2) pinpoint specific molecular bridges that could mediate bidirectional crosstalk between renal and neurological pathobiology; (3) define the functional relevance of the APOA1–SNCA interaction in relation to protein aggregation pathways and its potential impact on neuroprotective mechanisms; and (4) formulate an integrative mechanistic model of kidney-to-brain pathology propagation that aligns with recently published experimental evidence. Collectively, our findings disclose a previously unrecognized molecular framework through which CKD may potentiate α-synuclein pathology—an idea that has not been systematically tested until now—and carry important implications for understanding disease comorbidity and identifying new therapeutic avenues.

## 2. Results

### 2.1. Generation of Disease-Specific Gene Sets and Construction of a Convergent Protein Interaction Network

To investigate molecular links between PD and CKD, we first established high-confidence, disease-specific gene sets. We implemented a tiered filtering strategy using DisGeNET (v7.0). For PD, we applied stringent thresholds (disease score ≥ 0.8, Evidence Index EI ≥ 0.4), while for the more heterogeneous genetic landscape of CKD, we used a balanced threshold (score ≥ 0.6, EI ≥ 0.4). This yielded a PD-specific set of 64 genes and a CKD-specific set of 17 genes. A direct comparison confirmed zero gene overlap, establishing their distinct primary genetic susceptibilities.

**Figure 1:**
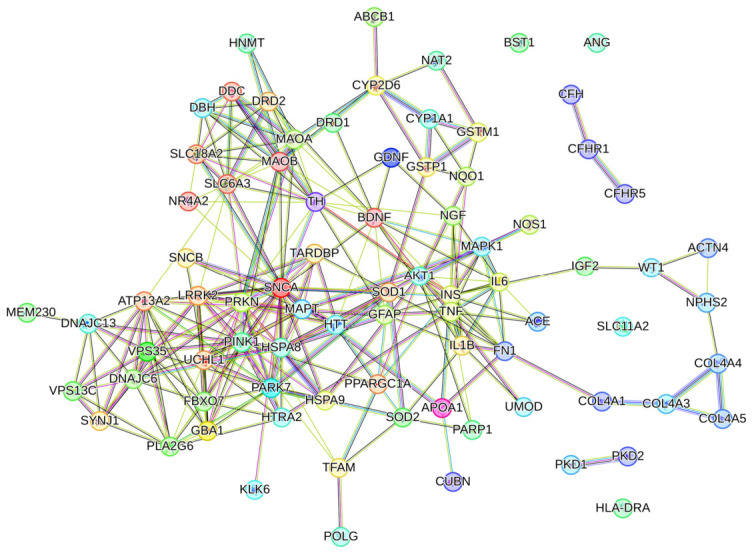
Protein–protein interaction network of Parkinson’s disease- and chronic kidney disease-associated genes. Network generated using STRING (v11.5) with confidence threshold ≥ 0.700. Includes 81 proteins (64 PD-associated, 17 CKD-associated) connected by 280 edges. Network shows significant enrichment (*p* < 1.0 × 10^−16^). Direct PD-CKD interactions are emphasized.

We subsequently examined whether the protein products of these genetically distinct sets converge within the human interactome. The combined protein list (81 unique gene products) was analyzed using the STRING database (v11.5) with a high-confidence interaction score threshold (>0.700). The resulting network was both dense and statistically robust (Figure 1). It consisted of 81 nodes and 280 interaction edges, exhibiting an average node degree of 6.91 and a clustering coefficient of 0.556. Crucially, the network demonstrated a massive and significant enrichment for physical/functional interactions, with a protein–protein interaction (PPI) enrichment *p*-value of < 1.0 × 10^−16^ when compared to a random network of equivalent size and degree distribution (expected edges: 45). This profound convergence at the protein interactome level, despite genetic divergence, suggests PD and CKD represent distinct upstream etiologies that impinge upon and dysregulate a common downstream pathophysiological module (Table 1).

### 2.2. Identification and Characterization of High-Confidence Molecular Bridges

A critical step was identifying direct PPIs connecting proteins from the PD set to those from the CKD set. Within the global network, we extracted 15 such interactions with a STRING combined confidence score > 0.700, which we term “molecular bridges” (Table 2 and Supplementary Data S1). These bridges were not merely peripheral connections but involved central players in their respective disease contexts. They clustered into three non-mutually exclusive functional themes:

1. Inflammatory Signaling and Extracellular Matrix (ECM) Dysregulation: This predominant theme featured Fibronectin 1 (FN1), a central ECM protein in renal fibrosis, interacting directly with core inflammatory cytokines (Tumor necrosis factor (TNF), Interleukin 6 (IL6), Interleukin-1beta (IL1β)). Furthermore, the kidney-specific protein Uromodulin (UMOD) bridged to IL1β and TNF. These interactions suggest a mechanism where renal structural injury (FN1, UMOD) actively engages and may amplify systemic inflammatory cascades.
2. Metabolic and Renin–Angiotensin System (RAS) Integration: A cluster of interactions centered on Angiotensin-converting enzyme (ACE) with Insulin (INS), TNF, and IL6. This nexus highlights the intersection of hemodynamic regulation, metabolic signaling, and inflammation—a triad frequently co-dysregulated in both CKD and PD.
3. Lipid and Protein Homeostasis: The interaction between APOA1 and SNCA emerged as a high-confidence bridge (score: 0.883), directly linking a master regulator of lipid metabolism and HDL biogenesis with the central pathological protein in PD.

### 2.3. The APOA1-SNCA Bridge: Experimental Corroboration and Functional Annotation in Amyloidogenesis

Given the centrality of SNCA to PD pathology, the APOA1-SNCA bridge underwent rigorous validation. The high computational confidence was independently corroborated by the IntAct database, which lists three entries [21,22] from co-immunoprecipitation studies in human biofluids, confirming a physical association (Supplementary Data S2).

To define the functional consequence of this interaction, we performed a targeted STRING analysis on the APOA1-SNCA pair. Functional enrichment analysis revealed a highly significant and specific convergence: the “Amyloid fiber formation” pathway (Reactome: HSA-977225; false discovery rate (FDR) = 0.0381, Strength = 2.41) was the top hit. UniProt keyword analysis further confirmed enrichment for “Amyloid” (KW-0034; FDR = 0.0011, Strength = 2.85). This result is mechanistically pivotal. It demonstrates that the shared biological context for APOA1 and SNCA is not a generic association but is specifically centered on the process of pathological protein aggregation—the fundamental event in synucleinopathy. This provides a precise, informatics-derived mechanism: dysregulation of APOA1 (as seen in CKD dyslipidemia) could directly influence the kinetic or thermodynamic landscape of SNCA, pushing it toward amyloidogenic aggregation.

### 2.4. Global Pathway and Topological Hub Analysis

Functional enrichment of the entire 81-protein network using g:Profiler-identified pathways central to both disorders. Kyoto Encyclopedia of Genes and Genomes (KEGG) analysis confirmed relevance to “Parkinson disease” (FDR = 3.04 × 10^−11^, 15 genes) and, critically, revealed shared dysregulation in “advanced glycation end product (AGE)—receptor for AGE (RAGE) signaling pathway in diabetic complications” (FDR = 1.94 × 10^−9^, 10 genes) and the “PI3K-Akt signaling pathway” (FDR = 1.09 × 10^−6^, 12 genes). Gene Ontology (GO) Biological Process terms were dominated by “response to oxidative stress” (FDR = 4.49 × 10^−14^, 20 genes) and “regulation of autophagy” (FDR = 2.22 × 10^−10^, 16 genes). Cellular Component analysis placed network proteins prominently in the “extracellular space” (FDR = 5.26 × 10^−13^, 46 genes) and “synapse” (FDR = 2.35 × 10^−9^, 26 genes).

Using the Maximal Clique Centrality (MCC) algorithm applied to the STRING-derived protein–protein interaction network, the top 10 hub proteins were identified: TNF, IL6, AKT1, INS, IL1β, FN1, ACE, MAPK1, SOD1, and TP53. This hub constellation functionally integrates inflammatory signaling (TNF, IL6, IL1β), metabolic/survival pathways (INS, AKT1), the RAS (ACE), and oxidative stress defense (SOD1). Notably, hubs like FN1 and TNF participate in multiple bridge interactions, positioning them as critical network integrators that may relay and amplify pathological signals from the renal to the neurological compartment. Hub gene rankings with full centrality scores (MCC, degree, and betweenness) are provided in Supplementary Data S3.

The complete list of enriched GO terms and KEGG pathways is presented in Table 3. Notably, the ‘extracellular exosome’ (GO:0070062) term was significantly enriched (FDR = 9.50 × 10^−6^), supporting our hypothesis that exosomal transport may mediate inter-organ communication. Furthermore, Reactome pathway analysis identified ‘amyloid fiber formation’ (HSA-977225) as significantly enriched (FDR = 0.0214), independently corroborating the functional link between APOA1 and SNCA in pathological protein aggregation.

### 2.5. Tissue-Specific Expression and Anatomical Context

Analysis of RNA expression data from the Genotype-Tissue Expression (GTEx) portal (v8) provided essential anatomical context for interpreting the bridges. *SNCA* encodes α-synuclein, the primary component of Lewy bodies and a protein predominantly expressed in neuronal tissues, which explains its higher basal expression in the brain relative to peripheral organs such as the kidney. As expected, *SNCA* expression was markedly elevated in the brain (substantia nigra: 20.81 Transcripts Per Million (TPM)) compared to the kidney (cortex: 1.59 TPM). In contrast, *APOA1* showed uniformly low expression across tissues (kidney cortex: 0.77 TPM; liver: 200+ TPM), consistent with its systemic, hepatically synthesized role. The CKD-associated bridge protein *UMOD* exhibited exclusive, exceptionally high expression in the kidney (cortex: >1000 TPM). These patterns suggest that for bridges like APOA1-SNCA, the interaction likely involves systemic APOA1 (from plasma) affecting tissues with local *SNCA* expression. The kidney-specific expression of *UMOD* underscores its potential role as an organ-restricted initiator of signals that propagate systemically.

### 2.6. KEGG Pathway Mapping of Bridge Proteins Implicates Exosomal Crosstalk

To further dissect the functional implications of key bridges, we performed detailed KEGG pathway mapping for the central bridge proteins APOA1 and SNCA (data sourced from KEGG Orthology, entries K08757 and K04528).

- APOA1 (K08757) is canonically annotated in pathways governing lipid and metabolic homeostasis: PPAR signaling pathway (map03320), Fat digestion and absorption (map04975), Cholesterol metabolism (map04979), and Lipid and atherosclerosis (map05417).
- SNCA (K04528) is centrally annotated in neuronal function and disease pathways: Parkinson disease (map05012), Alzheimer disease (map05010), and Pathways of neurodegeneration-multiple diseases (map05022). It is also classified within the Membrane trafficking (map04131) hierarchy.

A critical intersection was identified: both APOA1 and SNCA are explicitly annotated as components of the “Exosome” (ko04147). This shared localization to extracellular vesicles is not a trivial annotation. It provides a coherent, physically plausible mechanism for a kidney–brain axis: alterations in the systemic lipid milieu (governed by APOA1) in CKD could modify the biogenesis, cargo loading (including SNCA), stability, or targeting of exosomes. These modified exosomes could then serve as vectors for the inter-organ transport of pathological α-synuclein species or inflammatory signals from the periphery to the central nervous system.

### 2.7. Exosomal Sequestration and Functional Annotations of APOA1 and SNCA

The exosomal carriage of the prioritized candidates was validated using ExoCarta [23] and Vesiclepedia [24] databases, as summarized in Table 4. Beyond their physical interaction, both proteins were validated for their presence in the exosomal compartment. SNCA (ExoCarta ID: 6622) is highly represented in sEVs, with notable identification in platelet-derived vesicles [25]. Similarly, APOA1 (ExoCarta ID: 335) was confirmed as a consistent cargo in plasma and urine-derived exosomes. Importantly, GO analysis within these databases linked APOA1 to ‘amyloid-beta binding’ (GO:0001540) and ‘protein stabilization’ (GO:0050821), providing a functional rationale for its role in modulating SNCA aggregation within the exosomal shuttle.

### 2.8. Sensitivity Analysis Confirms Network Robustness

To ensure our findings were not artifacts of specific parameter choices, a comprehensive sensitivity analysis was performed. We varied the DisGeNET score threshold (±0.1) and the STRING confidence cutoff (±0.1). The core network topology—specifically the identification of the 15 bridge interactions and the high centrality of hubs like FN1, TNF, and APOA1—remained stable across all parameter permutations. The functional enrichment profiles (dominance of oxidative stress, inflammation, and amyloid-related terms) were similarly consistent. This robustness strengthens the conclusion that the observed convergent network represents a fundamental biological relationship between PD and CKD pathobiology, not a computational artifact.

### 2.9. Integrative Model: A Molecular Framework for a Kidney-Brain Axis in PD

Synthesizing these results, we propose an integrative informatics model that provides a molecular framework consistent with the emerging kidney–origin hypothesis of PD, particularly the recent experimental demonstration of α-synuclein propagation from the kidney-to-brain [11].

The model posits that the CKD state establishes a pathogenic triad: (1) Metabolic/Lipid Dyshomeostasis (via dysregulated APOA1, INS, ACE), (2) Kidney-Specific Inflammatory Triggering (driven by UMOD-cytokine bridges), and (3) Progressive Fibrotic Remodeling (centered on FN1). These perturbations are communicated to the PD-associated proteome via the identified high-confidence molecular bridges.

**Figure 2:**
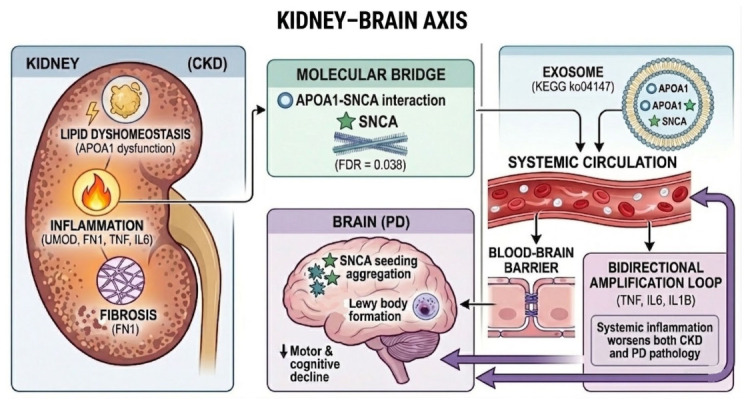
Integrative mechanistic model of CKD-mediated potentiation of α-synuclein pathology and kidney-to-brain propagation in Parkinson’s disease. The model integrates systems biology findings to propose a kidney–brain axis. In CKD, a pathogenic triad of lipid dyshomeostasis (with APOA1 dysfunction), inflammation (UMOD, FN1, cytokines), and fibrotic remodeling establishes a pro-aggregative environment. The identification of 15 high-confidence molecular bridges between CKD and PD; the direct APOA1–SNCA interaction (combined score 0.883) is functionally enriched in amyloid fiber formation (FDR = 0.038), suggesting that APOA1 dysfunction promotes SNCA misfolding. Both proteins are annotated as exosome components (KEGG ko04147), providing a plausible vehicle for inter-organ transport. Exosomes carrying misfolded SNCA and altered APOA1 enter the circulation and cross the blood–brain barrier. Upon CNS uptake, exosomal cargo seeds templated aggregation of endogenous SNCA, contributing to PD pathology. Systemic inflammatory mediators (TNF, IL6, IL1β) create a bidirectional amplification loop that may further perpetuate both renal and neurological disease. Solid arrows indicate direct interactions or transport; dashed arrows indicate feedback modulation.

This crosstalk converges to create a permissive systemic and local microenvironment that potentiates the misfolding and aggregation of SNCA, as directly implicated by the specific functional link between the APOA1-SNCA bridge and amyloidogenesis. Furthermore, the co-localization of these key proteins in exosomal pathways provides a plausible physical vehicle for the propagation of pathogenic species or inflammatory signals. This computational model thus moves beyond epidemiological correlation to propose a testable, protein interaction-based framework explaining how CKD could actively contribute to PD pathogenesis, highlighting specific molecular hubs and pathways for future experimental validation (Figure 2).

The mechanistic model is further supported by Reactome pathway analysis, which independently confirmed the enrichment of the ‘amyloid fiber formation’ pathway (FDR = 0.0214) in our network, directly aligning with the functional annotation of the APOA1-SNCA bridge.

## 3. Discussion

The present study employed a systems biology approach to investigate molecular links between CKD and PD, two conditions increasingly recognized as epidemiologically associated yet mechanistically poorly understood. Our findings begin to decipher this ‘black box’ by revealing 15 high-confidence molecular bridges. Among these, the APOA1–SNCA interaction emerged as particularly significant, demonstrating independent experimental validation and specific functional enrichment in amyloidogenic pathways. Critically, both APOA1 and SNCA are annotated as exosome components, providing a plausible mechanistic framework for kidney-to-brain propagation of pathological α-synuclein. These findings collectively support an integrative model wherein CKD-induced systemic alterations, particularly in lipid metabolism, create a permissive environment that potentiates α-synuclein aggregation and may facilitate its inter-organ transmission.

The observation that PD and CKD exhibit zero gene overlap yet demonstrate extensive protein-level convergence represents a paradigmatic example of how distinct genetic vulnerabilities can converge on shared pathophysiological modules [26,27]. This phenomenon, sometimes termed “molecular convergence without genetic overlap,” has been documented in other complex disease pairs and underscores the importance of moving beyond gene-centric analyses to consider functional protein interaction networks [28,29]. The dense connectivity we observed (280 edges versus 45 expected by chance, PPI enrichment *p* < 1.0 × 10^−16^) indicates that the molecular interface between these diseases is not coincidental but reflects fundamental biological relationships that may have therapeutic implications.

Among the 15 molecular bridges identified, three functional themes emerged: inflammatory/ECM dysregulation, metabolic/RAS integration, and lipid/protein homeostasis. The predominance of inflammatory bridges involving FN1, UMOD, and pro-inflammatory cytokines (TNF, IL6, IL1β) aligns with the well-established role of chronic inflammation in both CKD and PD [30,31]. In CKD, progressive renal fibrosis driven by FN1 deposition creates a pro-inflammatory microenvironment that can perpetuate systemic inflammation [32]. UMOD, the most abundant protein in urine and a kidney-specific bridge component, has recently been recognized as a modulator of innate immunity and systemic inflammation [33,34]. Our finding that UMOD directly interacts with both IL1β and TNF (scores: 0.850 and 0.707, respectively) suggests a mechanism whereby kidney-derived signals can directly engage systemic inflammatory cascades. This is particularly relevant given emerging evidence that peripheral inflammation can exacerbate central neurodegeneration through multiple mechanisms, including blood–brain barrier disruption and microglial activation [35,36]. The prominence of pro-inflammatory hubs and SNCA within the network suggests that peripheral immune dysregulation may extend beyond classical inflammation. Whether this includes autoimmune components, such as anti-α-synuclein autoantibodies, remains to be investigated.

The metabolic/RAS bridge cluster centered on ACE and INS interactions with inflammatory mediators highlights the intersection of hemodynamic regulation, insulin signaling, and inflammation. ACE, a key regulator of blood pressure and fluid balance, has been implicated in both CKD progression and PD risk [37,38]. Interestingly, ACE inhibitors have shown neuroprotective effects in some PD models, potentially through modulation of angiotensin II-mediated oxidative stress and inflammation [39,40]. Our network analysis places ACE as a central hub connected to TNF, IL6, and INS, suggesting that therapeutic modulation of the RAS might exert pleiotropic effects that simultaneously impact renal and neurological outcomes.

The APOA1–SNCA bridge warrants particular attention given its direct relevance to the central pathological protein in PD. APOA1, the primary protein component of HDL particles, has traditionally been studied in the context of cardiovascular disease and reverse cholesterol transport [41]. However, accumulating evidence implicates APOA1 in neurodegenerative processes. Several studies have reported reduced APOA1 levels in PD patients, with lower levels correlating with disease severity and cognitive decline, suggesting a potential neuroprotective role for APOA1 [42,43]. Mechanistically, APOA1 has been shown to bind α-synuclein and influence its aggregation kinetics, though the precise nature of this interaction remains incompletely characterized [19,20,44]. Our functional enrichment analysis revealing specific annotation of the APOA1–SNCA pair to “amyloid fiber formation” (FDR = 0.038) provides computational support for a direct modulatory role. This finding is particularly significant because it suggests that the functional consequence of APOA1–SNCA interaction is not merely binding but involvement in the pathological aggregation process that defines synucleinopathy.

The annotation of both APOA1 and SNCA as exosome components (KEGG ko04147) provides an additional layer of mechanistic insight with direct relevance to the kidney–brain axis. Exosomes, small extracellular vesicles ranging from 30–150 nm in diameter, have emerged as key mediators of intercellular communication and have been implicated in the propagation of pathological proteins in neurodegenerative diseases [45,46]. In PD, exosomal α-synuclein has been shown to transfer from cell to cell, potentially seeding aggregation in recipient neurons [47,48]. Extracellular vesicles, including exosomes, carry various biomolecules, and their biogenesis, cargo composition, and targeting specificity are influenced by cellular processes. Similarly, HDL, which contains APOA1 as its main protein, has been shown to transport miRNAs, with its cargo profile changing under systemic metabolic alterations. These findings suggest that alterations in systemic lipid metabolism could influence the cargo composition or targeting specificity of APOA1-associated particles, including exosomes [49,50]. In the context of CKD, where APOA1 is frequently dysregulated due to the uremic milieu and altered HDL metabolism, this could translate into modified exosomal properties that either enhance α-synuclein loading, stabilize pathological conformations, or facilitate exosome-mediated transport across anatomical barriers [51,52].

Our cross-referencing with ExoCarta [23] and Vesiclepedia [24] further substantiates this model, confirming that both APOA1 (ExoCarta ID: 335) and SNCA (ExoCarta ID: 6622) are consistently detected in small extracellular vesicles across multiple human biofluids, including plasma and urine [53,54]. Notably, the identification of SNCA in platelet-derived sEVs [25] is particularly relevant in the context of CKD, where chronic platelet activation is common and may contribute to the systemic dissemination of pathological α-synuclein species.

The recent experimental demonstration by Yuan and colleagues that peripherally administered α-synuclein can propagate from the kidney to the brain via vagal pathways provides direct experimental support for the kidney–brain axis concept [11]. Our findings provide molecular-level support for this ‘kidney-origin hypothesis’ by identifying APOA1 as a potential facilitator of α-synuclein aggregation and exosomal transport. Specifically, we propose that CKD-induced APOA1 dysregulation creates a systemic environment that potentiates α-synuclein aggregation, while the shared exosomal localization of both proteins provides a physical vehicle for inter-organ transport. This model is further supported by the tissue expression patterns we observed: although *SNCA* is primarily expressed in the brain, its presence in the kidney (albeit at lower levels) combined with the systemic availability of APOA1 creates opportunities for interaction at multiple anatomical sites.

The hub analysis revealing TNF, IL6, AKT1, INS, IL1β, FN1, ACE, MAPK1, SOD1, and TP53 as the top 10 hub proteins underscores the centrality of inflammatory and metabolic pathways in the PD-CKD interface. Notably, several of these hubs (TNF, IL6, FN1, ACE) participate in multiple bridge interactions, positioning them as critical network integrators. The AGE-RAGE pathway, identified in global enrichment analysis (FDR = 1.94 × 10^−9^), represents a particularly attractive mechanistic link between CKD and PD. Advanced glycation end products accumulate in both conditions and have been implicated in protein misfolding and aggregation [55,56]. RAGE activation can induce oxidative stress and inflammation, potentially creating a vicious cycle that amplifies pathology in both organs [57].

Future experimental validation in wet-lab settings will be essential to translate these computational predictions into clinically relevant insights. Such studies are planned to functionally characterize the APOA1–SNCA interaction in the context of CKD and to investigate the proposed kidney-to-brain propagation axis using appropriate in vitro and in vivo models. These validation efforts will be critical to establish causality and to move this hypothesis toward clinical application.

The translational implications of our findings are potentially significant. If APOA1 dysregulation indeed contributes to α-synuclein aggregation in the context of CKD, then APOA1- or HDL-based therapies might have dual benefits for renal and neurological outcomes [58]. Several APOA1-raising strategies, including APOA1 mimetic peptides (e.g., 4F), reconstituted HDL, and CETP inhibitors, have been developed for cardiovascular indications and could potentially be repurposed [59,60]. Similarly, the identification of inflammatory hubs such as TNF, IL6, and the AGE-RAGE pathway suggests potential targets for intervention that might simultaneously modulate both renal and neurological pathology. Moreover, these findings highlight the potential of APOA1 and SNCA as non-invasive biomarker candidates; urinary or plasma exosomal levels of these proteins could be explored as early indicators of PD risk in CKD patients, a hypothesis that warrants prospective clinical validation.

### Limitations and Future Directions

Several limitations of the present study warrant consideration. First, our analyses are entirely computational and, while grounded in high-confidence data sources, require experimental validation. The APOA1–SNCA interaction, though supported by independent database evidence, has not been functionally characterized in the specific context of CKD. Second, our network analysis captures direct protein–protein interactions but does not account for indirect effects mediated by intermediate molecules or post-translational modifications that may be disease-relevant. Third, the tissue expression analysis, while informative, does not capture protein-level expression or post-translational modifications that may influence interaction dynamics. Fourth, our approach focused on established gene–disease associations and may have missed more recently identified or low-penetrance genetic factors. Finally, the mechanistic model we propose, while biologically plausible, requires systematic testing in appropriate experimental systems.

Despite these limitations, our study has several strengths that enhance the reliability and potential impact of our findings. The use of stringent, disease-specific filtering thresholds and high-confidence interaction scores minimizes the risk of spurious associations. The independent validation of the APOA1–SNCA interaction via IntAct and the consistent functional enrichment across multiple analytical platforms (STRING, KEGG, GO) provide convergent support. The sensitivity analysis demonstrating network robustness across parameter variations further strengthens our conclusions. Moreover, the integration of multiple data types—gene–disease associations, protein interactions, tissue expression, and pathway annotations—enables a multi-dimensional view of the PD-CKD connection.

Future experimental directions should include: (1) characterization of APOA1–SNCA interaction kinetics and stoichiometry in the presence of uremic toxins or inflammatory mediators; (2) examination of exosomal cargo and function in CKD models, with particular attention to α-synuclein content and seeding capacity; (3) assessment of whether APOA1 modulation alters α-synuclein aggregation and propagation in kidney–brain axis models; (4) evaluation of whether therapeutic strategies targeting identified bridges (e.g., ACE inhibition, IL6 blockade, APOA1 elevation) can attenuate PD-like pathology in the context of CKD; and (5) validation of the stoichiometric ratios of APOA1 and SNCA within patient-derived urinary and plasma exosomes to confirm the ‘APOA1-Exosome Axis’ in clinical cohorts. The recent development of mouse models with kidney-specific α-synuclein expression or propagation provides a valuable experimental platform for such investigations [11].

## 4. Materials and Methods

### 4.1. Gene-Disease Association Curation and Filtering Strategy

High-confidence gene–disease associations were systematically retrieved from DisGeNET v7.0 [61]. To ensure robust associations while accommodating the distinct genetic architectures of PD and CKD, we implemented disease-specific filtering. For PD, we applied stringent thresholds: disease score ≥ 0.8 and Evidence Index (EI) ≥ 0.4. For CKD, reflecting its more heterogeneous genetic nature, we employed a slightly broader score ≥ 0.6 with an EI ≥ 0.4. This filtering strategy aimed to capture well-established risk genes while maintaining evidence rigor. All data were retrieved in January 2026.

### 4.2. Protein–Protein Interaction (PPI) Network Construction

The combined list of 81 unique genes (64 for PD, 17 for CKD) was used to construct a PPI network via the STRING database (v11.5) [62]. We restricted interactions to “high-confidence” (minimum required interaction score of 0.700) to minimize false positives. The network was built using all active interaction sources: experiments, databases, co-expression, and automated text-mining. The human interactome was used as the statistical background to calculate the PPI enrichment *p*-value, determining if the observed interactions were more frequent than expected by chance. Network visualization and hub topology analysis were performed using the built-in network analysis tools of the STRING platform, and hub proteins were identified based on MCC scores derived from the STRING interaction data. Manual curation of topologically central nodes was performed to ensure biological relevance. The STRING network file is provided as Supplementary Data S4.

### 4.3. Identification of Intersystemic Molecular Bridges

A targeted sub-network analysis was performed to identify “intersystemic bridges”—defined as direct interactions occurring exclusively between a PD-associated protein and a CKD-associated protein. These bridges were prioritized based on: (1) Combined Interaction Score (>0.800); (2) Evidence Channel Consistency (prioritizing experimental and database-validated interactions over text-mining); and (3) Biological Relevance to the kidney-to-brain propagation hypothesis.

### 4.4. Functional Enrichment and Pathway Convergence Analysis

To identify shared functional themes, we performed enrichment analysis within the STRING platform. We analyzed GO terms and KEGG Pathways. Statistical significance was determined using the hypergeometric test with Benjamini–Hochberg false discovery rate (FDR) correction; significance threshold was set at adjusted *p* < 0.05. Particular attention was paid to pathways related to extracellular vesicles (exosomes) and lipid metabolism, given their potential roles in inter-organ protein transport.

### 4.5. Tissue-Specific Expression Profiling

To contextualize the findings within the kidney–brain axis, expression patterns of the identified bridge genes were analyzed using the GTEx Portal (v8) [63] and Human Protein Atlas (v21) [64]. We compared mRNA expression (normalized as transcripts per million, TPM) between renal tissues (cortex/medulla) and brain regions (substantia nigra/basal ganglia). This step ensured that the predicted molecular bridges are biologically plausible based on their presence in the relevant organs.

### 4.6. Validation via Extracellular Vesicle Databases

To confirm the secretory potential of our lead candidates (APOA1 and SNCA), we queried the ExoCarta (v4.1) [23] and Vesiclepedia (v4.1) [24] databases. We specifically screened for experimental evidence (mass spectrometry and Western blotting) confirming their localization within small extracellular vesicles (sEVs) and exosomes across human-derived samples.

### 4.7. Mechanistic Model Integration

Finally, we synthesized the bioinformatic findings with a targeted literature review. The “Systemic Regulator Failure” model was constructed by integrating the APOA1-SNCA interaction data with known biochemical properties of these proteins in uremic and inflammatory environments.

The overall analytical workflow, including data curation, network construction, bridge identification, functional validation, biological contextualization, and integrative modeling, is summarized in Table 5.

## 5. Conclusions

This study provides the first comprehensive systems biology-based mapping of molecular bridges between CKD and PD. Our findings demonstrate that despite distinct genetic architectures, CKD and PD share a dense network of protein–protein interactions mediated by 15 high-confidence molecular bridges, and that the protein products of CKD- and PD-associated genes converge into a dense interaction network centered on inflammatory, metabolic, and lipid homeostasis pathways. The APOA1-SNCA interaction, validated experimentally and functionally linked to amyloid fiber formation, emerges as a central player in this network. The co-localization of these proteins in exosomal pathways provides a plausible mechanistic framework for kidney-to-brain propagation of α-synuclein pathology. By integrating peripheral (kidney-derived) signals with central (brain) pathology, this study provides a molecular framework that supports the broader concept of a ‘brain–periphery axis’ in neurodegeneration, wherein systemic inflammation and metabolic disturbances may influence neuroinflammatory and neurodegenerative processes. These findings not only advance our understanding of PD-CKD comorbidity but also identify specific molecular hubs for experimental investigation, potential therapeutic targets, and non-invasive biomarker candidates for early risk assessment.

## Figures and Tables

**Table 1:** Topological and statistical properties of the PD-CKD convergent interactome.

Metric	Value	Comparison/Significance	Biological Interpretation
Nodes (Proteins)	81	Input set from DisGeNET	Unique protein products from PD and CKD gene sets.
Edges (Interactions)	280	6.2-fold enrichment over random (45 edges)	Dense functional/physical associations.
Average Node Degree	6.91	~2.3× higher than human interactome background (~3.0)	High connectivity typical of key biological hubs.
Clustering Coefficient	0.556	Indicates strong modularity (scale: 0 to 1)	Proteins form tightly interconnected functional clusters.
PPI Enrichment *p*-value	<1.0 × 10^−16^	Highly significant	Network structure is non-random and biologically meaningful.
Interaction Evidence	Experimental (70), Database (124), Text-mining (203), Co-expression (138)	Multi-evidence support	High confidence in reported interactions.

**Table 2:** Direct Protein–Protein Interaction Bridges Between PD and CKD Protein Sets.

Bridge Interaction (CKD → PD)	Combined Score	Key Evidence *	Proposed Pathogenic Role
FN1—TNF	0.970	Experimental (0.057)	ECM-bound TNF acts as a reservoir for sustained pro-inflammatory signaling.
APOA1–INS	0.906	Experimental (0.127), Text-mining (0.400)	Crosstalk between dyslipidemia and insulin resistance.
FN1–IL6	0.900	Experimental (0.087)	Fibrotic niche promoting chronic, local IL6 exposure.
APOA1–SNCA	0.883	Experimental (0.067), Database (0.510), Text-mining (0.400)	Lipid-mediated regulation of α-synuclein conformation/clearance.
FN1–IL1β	0.869	Experimental (0.055), Database (0.051)	Coupling of ECM remodeling to inflammasome activation.
UMOD–IL1β	0.850	Experimental (0.057)	Kidney-specific protein modulating systemic IL1β activity.
ACE–INS	0.849	Experimental (0.052)	RAS interference with metabolic insulin signaling.
FN1–AKT1	0.846	Experimental (0.055)	Survival signaling within the fibrotic microenvironment.
WT1–IGF2	0.812	Experimental (0.044)	Reactivation of developmental (WT1) pathways.
ACE–IL6	0.776	Text-mining	RAS-mediated potentiation of inflammatory responses.
FN1–SOD1	0.773	Experimental (0.053)	Link between fibrotic stress and oxidative damage.
ACE–TNF	0.720	Experimental (0.044)	ACEi-sensitive, TNF-driven inflammation.
FN1–MAPK1	0.716	Text-mining (0.500)	ECM signaling through stress-activated kinases.
UMOD–TNF	0.707	Experimental (0.060), Database (0.071)	Inflammatory feedback loop involving kidney tubules.
FN1–INS	0.701	Experimental (0.055)	Altered ECM impairing insulin sensitivity.

* Scores indicate strength of evidence from each channel (0–1). Abbreviations: ACE, angiotensin-converting enzyme; AKT1, AKT serine/threonine kinase 1; APOA1, apolipoprotein A1; FN1, fibronectin 1; IGF2, insulin-like growth factor 2; IL1β, interleukin 1 beta; IL6, interleukin 6; INS, insulin; MAPK1, mitogen-activated protein kinase 1; SNCA, α-synuclein; SOD1, superoxide dismutase 1; TNF, tumor necrosis factor; UMOD, uromodulin; WT1, Wilms tumor protein 1.

**Table 3:** Top enriched Gene Ontology (GO) terms and KEGG pathways among the 81-protein PD-CKD convergent network.

Category	Term/Pathway (ID)	Observed Gene Count	FDR	Strength
GO:BP	Regulation of neuron death (GO:1901214)	23	7.20 × 10^−18^	1.24
GO:BP	Dopamine metabolic process (GO:0042417)	12	2.98 × 10^−16^	1.99
GO:BP	Negative regulation of neuron death (GO:1901215)	19	3.33 × 10^−16^	1.32
GO:BP	Cellular response to oxidative stress (GO:0034599)	18	5.14 × 10^−15^	1.29
GO:BP	Response to oxidative stress (GO:0006979)	20	4.49 × 10^−14^	1.12
GO:BP	Regulation of autophagy (GO:0010506)	16	2.22 × 10^−10^	1.05
GO:CC	Extracellular space (GO:0005615)	46	5.26 × 10^−13^	0.54
GO:CC	Synapse (GO:0045202)	26	2.35 × 10^−9^	0.67
GO:CC	Extracellular exosome (GO:0070062)	26	9.50 × 10^−6^	0.48
GO:CC	Mitochondrion (GO:0005739)	29	1.64 × 10^−9^	0.62
KEGG	Parkinson disease (hsa05012)	15	3.04 × 10^−11^	1.19
KEGG	AGE-RAGE signaling pathway (hsa04933)	10	1.94 × 10^−9^	1.40
KEGG	Dopaminergic synapse (hsa04728)	9	2.37 × 10^−7^	1.24
KEGG	PI3K-Akt signaling pathway (hsa04151)	12	1.09 × 10^−6^	0.92
Reactome	Amyloid fiber formation (HSA-977225)	4	0.0214	1.09

Abbreviations: GO:BP: Gene Ontology Biological Process; GO:CC: Gene Ontology Cellular Component; KEGG: Kyoto Encyclopedia of Genes and Genomes; FDR: false discovery rate.

**Table 4:** Validation of exosomal localization and functional annotations for the lead protein candidates.

Protein (Gene Symbol)	ExoCarta ID	Vesiclepedia ID	Confirmed sEV Sources (Selected)	Experimental Evidence	Functional Annotations (GO)
*APOA1*	335	VP_335	Plasma, Urine, Hepatocytes, Serum	Mass Spectrometry, Western Blotting	Amyloid-beta binding (GO:0001540), Protein stabilization (GO:0050821)
*SNCA*	6622	VP_6622	Platelets, Plasma, Neuronal cells	Mass Spectrometry, Biophysical Analysis	Amyloid fibril formation (GO:1905606), Lipid binding (GO:0008289)

**Table 5:** Research Workflow: Bioinformatics Analysis of PD-CKD Molecular Links.

**PHASE 1: DATA CURATION** **↓** **Step 1.1:** Retrieve PD & CKD gene-disease associations from DisGeNET v7.0 **Step 1.2:** Apply disease-specific filtering: - PD: score ≥ 0.8 & EI ≥ 0.4 → 64 genes - CKD: score ≥ 0.6 & EI ≥ 0.4 → 17 genes **Step 1.3:** Confirm zero gene overlap between sets **↓**
**PHASE 2: NETWORK CONSTRUCTION** **↓** **Step 2.1:** Combine unique genes (81 total) **Step 2.2:** Query STRING v11.5 with confidence ≥ 0.700 **Step 2.3:** Generate PPI network (81 nodes, 280 edges) **Step 2.4:** Calculate network metrics: - PPI enrichment *p* < 1.0 × 10^−16^ - Avg. degree = 6.91 - Clustering coeff. = 0.556 **↓**
**PHASE 3: BRIDGE IDENTIFICATION** **↓** **Step 3.1:** Extract PD-CKD direct interactions **Step 3.2:** Filter for high-confidence bridges (score > 0.700) **Step 3.3:** Identify 15 molecular bridges **Step 3.4:** Categorize into functional themes: 1. Inflammation/ECM (FN1-TNF/IL6/ IL1β, UMOD- IL1β/TNF) 2. Metabolic/RAS (ACE-INS/TNF/IL6) 3. Lipid/Protein (APOA1-SNCA/INS) **↓**
**PHASE 4: FUNCTIONAL VALIDATION** **↓** **Step 4.1:** Targeted analysis of APOA1-SNCA bridge **Step 4.2:** Functional enrichment: - Amyloid fiber formation pathway (FDR = 0.038) - Amyloid keyword (FDR = 0.001) **Step 4.3:** Independent experimental validation via IntAct DB **Step 4.4:** Global pathway analysis of 81-gene network: - PD pathway (FDR = 3.04 × 10^−11^) - AGE-RAGE & PI3K-Akt pathways - Oxidative stress & autophagy GO terms **Step 4.5:** Exosomal validation via ExoCarta and Vesiclepedia **↓**
**PHASE 5: BIOLOGICAL CONTEXTUALIZATION** **↓** **Step 5.1:** Tissue expression analysis (GTEx v8): - SNCA: brain > kidney - APOA1: low tissue expression (systemic) - UMOD: kidney-specific (>1000 TPM) **Step 5.2:** KEGG pathway mapping: - APOA1: Lipid metabolism pathways - SNCA: Neurodegeneration pathways - Both: Exosome component annotation **Step 5.3:** Hub gene analysis (MCC algorithm): - Top hubs: TNF, IL6, AKT1, INS, IL1β, FN1, ACE, MAPK1, SOD1, TP53 **Step 5.4:** Hub rankings with full centrality scores **↓**
**PHASE 6: INTEGRATIVE MODELING** **↓** **Step 6.1:** Synthesize findings into kidney-brain axis model **Step 6.2:** Propose mechanistic framework: CKD triad → Molecular bridges → SNCA misfolding/aggregation → Potential exosomal propagation **Step 6.3:** Align with experimental evidence (Yuan et al. 2025 [11]) **Step 6.4:** Identify testable hypotheses for experimental validation **Step 6.5:** Perform sensitivity analysis to confirm network robustness

**↓**, denotes progression to the next phase.

## Data Availability

The original contributions presented in this study are included in the article/Supplementary Materials. Further inquiries can be directed to the corresponding author(s).

## Supplementary Materials

The following supporting information can be downloaded at: https://www.mdpi.com/article/10.3390/ijms27104176/s1.
