# Supplementary material for: The APOA1-SNCA Axis as a Molecular Bridge Between CKD and Parkinson’s Disease: A Systems Biology Model of Kidney-to-Brain Propagation via Exosomal Pathways
Source: Int J Mol Sci. 2026 May 8;27(10):4176. doi: 10.3390/ijms27104176 (PMC13207088; doi:10.3390/ijms27104176)
Supplement: Supplementary file 1 [file ijms-27-04176-s001.zip › Supplementary Data S1.pdf]

Supplementary Material

Supplementary Data S1. High-confidence direct molecular bridge interactions between Parkinson's disease and chronic kidney disease associated proteins.

#node1	node2	node1_string_id	node2_string_id	neighborhood_on_chromosome	gene_fusion	phylogenetic_cooccurrence	homology	coexpression	experimentally_determined_interaction	database_annotated	automated_textmining	combined_score
ACE	TNF	9606.ENSP00000290866	9606.ENSP00000398698	0	0	0	0	0.044	0	0	0.720	0.720
ACE	IL6	9606.ENSP00000290866	9606.ENSP00000385675	0	0	0	0	0	0	0	0.776	0.776
ACE	INS	9606.ENSP00000290866	9606.ENSP00000380432	0	0	0	0	0.052	0	0	0.847	0.849
AKT1	FN1	9606.ENSP00000451828	9606.ENSP00000346839	0	0	0	0	0.055	0	0	0.844	0.846
APOA1	SNCA	9606.ENSP00000236850	9606.ENSP00000500990	0	0	0	0	0.067	0.510	0.400	0.625	0.883
APOA1	INS	9606.ENSP00000236850	9606.ENSP00000380432	0	0	0	0	0.127	0	0.400	0.835	0.906
FN1	MAPK1	9606.ENSP00000346839	9606.ENSP00000215832	0	0	0	0	0	0	0.500	0.455	0.716
FN1	IL1B	9606.ENSP0000034	9606.ENSP0000026	0	0	0	0	0.055	0.051	0	0.866	0.869

		6839	3341									
FN1	SOD1	9606.ENSP00000346839	9606.ENSP00000270142	0	0	0	0	0.053	0	0	0.771	0.773
FN1	INS	9606.ENSP00000346839	9606.ENSP00000380432	0	0	0	0	0.055	0	0	0.697	0.701
FN1	AKT1	9606.ENSP00000346839	9606.ENSP00000451828	0	0	0	0	0.055	0	0	0.844	0.846
FN1	IL6	9606.ENSP00000346839	9606.ENSP00000385675	0	0	0	0	0.087	0	0	0.895	0.900
FN1	TNF	9606.ENSP00000346839	9606.ENSP00000398698	0	0	0	0	0.057	0	0	0.970	0.970
IGF2	WT1	9606.ENSP00000391826	9606.ENSP00000368370	0	0	0	0	0.044	0	0	0.812	0.812
IL1B	UMOD	9606.ENSP00000263341	9606.ENSP00000379438	0	0	0	0	0.057	0	0	0.848	0.850
IL1B	FN1	9606.ENSP00000263341	9606.ENSP00000346839	0	0	0	0	0.055	0.051	0	0.866	0.869
IL6	ACE	9606.ENSP00000385675	9606.ENSP00000290866	0	0	0	0	0	0	0	0.776	0.776
IL6	FN1	9606.ENSP00000385675	9606.ENSP00000346839	0	0	0	0	0.087	0	0	0.895	0.900
INS	APOA1	9606.ENS	9606.ENS	0	0	0	0	0.127	0	0.400	0.835	0.906

		P00000380432	P00000236850									
INS	ACE	9606.ENSP00000380432	9606.ENSP00000290866	0	0	0	0	0.052	0	0	0.847	0.849
INS	FN1	9606.ENSP00000380432	9606.ENSP00000346839	0	0	0	0	0.055	0	0	0.697	0.701
MAPK1	FN1	9606.ENSP00000215832	9606.ENSP00000346839	0	0	0	0	0	0	0.500	0.455	0.716
SNCA	APOA1	9606.ENSP00000500990	9606.ENSP00000236850	0	0	0	0	0.067	0.510	0.400	0.625	0.883
SOD1	FN1	9606.ENSP00000270142	9606.ENSP00000346839	0	0	0	0	0.053	0	0	0.771	0.773
TNF	ACE	9606.ENSP00000398698	9606.ENSP00000290866	0	0	0	0	0.044	0	0	0.720	0.720
TNF	FN1	9606.ENSP00000398698	9606.ENSP00000346839	0	0	0	0	0.057	0	0	0.970	0.970
TNF	UMOD	9606.ENSP00000398698	9606.ENSP00000379438	0	0	0	0	0.060	0.071	0	0.691	0.707
UMOD	IL1B	9606.ENSP00000379438	9606.ENSP00000263341	0	0	0	0	0.057	0	0	0.848	0.850
UMOD	TNF	9606.ENSP00000379438	9606.ENSP00000398698	0	0	0	0	0.060	0.071	0	0.691	0.707

WT1	IGF2	9606.ENSP00000368370	9606.ENSP00000391826	0	0	0	0	0.044	0	0	0.812	0.812
