# Supplementary material for: The APOA1-SNCA Axis as a Molecular Bridge Between CKD and Parkinson’s Disease: A Systems Biology Model of Kidney-to-Brain Propagation via Exosomal Pathways
Source: Int J Mol Sci. 2026 May 8;27(10):4176. doi: 10.3390/ijms27104176 (PMC13207088; doi:10.3390/ijms27104176)
Supplement: Supplementary file 1 [file ijms-27-04176-s001.zip › Supplementary Data S2.pdf]

Supplementary Material

Supplementary Data S2. Experimentally validated physical interactions between SNCA and APOA1 from the IntAct database.

**Field**	**Interaction 1**	**Interaction 2**	**Interaction 3**
**Molecule A / B**	SNCA / APOA1	SNCA / APOA1	SNCA / APOA1
**Identifier A**	UniProt: P37840	UniProt: P37840	UniProt: P37840
**Identifier B**	UniProt: P02647	UniProt: P02647	UniProt: P02647
**Type A / B**	Protein / Protein	Protein / Protein	Protein / Protein
**Organism (Species)**	*Homo sapiens* / *Homo sapiens*	*Homo sapiens* / *Homo sapiens*	*Homo sapiens* / *Homo sapiens*
**Host Organism / Biofluid**	Blood plasma	Blood plasma	Cerebrospinal fluid (CSF)
**Detection Method**	Anti-bait co-immunoprecipitation	Anti-bait co-immunoprecipitation	Anti-bait co-immunoprecipitation
**Publication (PMID)**	28887769	28887769	31270237
**Interaction Type**	Association	Physical association	Association
**Interaction ID**	EBI-25297943	EBI-25297950	EBI-25295611
**Expansion Method**	Spoke expansion	—	Spoke expansion
**Aliases (A)**	NACP, Non-A beta component	NACP, Non-A beta component	NACP, Non-A beta component
**Aliases (B)**	Apolipoprotein A1, APOA1	Apolipoprotein A1, APOA1	Apolipoprotein A1, APOA1
