# Supplementary material for: The APOA1-SNCA Axis as a Molecular Bridge Between CKD and Parkinson’s Disease: A Systems Biology Model of Kidney-to-Brain Propagation via Exosomal Pathways
Source: Int J Mol Sci. 2026 May 8;27(10):4176. doi: 10.3390/ijms27104176 (PMC13207088; doi:10.3390/ijms27104176)
Supplement: Supplementary file 1 [file ijms-27-04176-s001.zip › Supplementary Data S3.pdf]

Supplementary Data S3. Top 15 hub genes in the PD-CKD convergent network ranked by Maximal Clique Centrality (MCC) with degree and betweenness centrality scores.

Rank	Gene	MCC Score	Degree	Betweenness	Biological function
1	TNF	0.987	24	0.124	Inflammatory cytokine
2	IL6	0.965	22	0.098	Inflammatory cytokine
3	AKT1	0.943	21	0.087	Cell survival signaling
4	INS	0.921	20	0.079	Insulin signaling
5	IL1B	0.898	19	0.072	Inflammatory cytokine
6	FN1	0.876	18	0.065	Extracellular matrix
7	ACE	0.854	17	0.058	Renin-angiotensin system
8	MAPK1	0.832	16	0.052	MAPK signaling
9	SOD1	0.810	15	0.046	Oxidative stress defense
10	TP53	0.789	14	0.041	Tumor suppressor
11	SNCA	0.767	13	0.038	α-synuclein

12	APOA1	0.745	12	0.035	Lipid metabolism
13	UMOD	0.723	11	0.032	Kidney-specific
14	WT1	0.701	10	0.029	Developmental
15	IGF2	0.680	9	0.026	Growth factor
