# Supplementary material for: The APOA1-SNCA Axis as a Molecular Bridge Between CKD and Parkinson’s Disease: A Systems Biology Model of Kidney-to-Brain Propagation via Exosomal Pathways
Source: Int J Mol Sci. 2026 May 8;27(10):4176. doi: 10.3390/ijms27104176 (PMC13207088; doi:10.3390/ijms27104176)
Supplement: Supplementary file 1 [file ijms-27-04176-s001.zip › Supplementary Data S4.pdf]

Supplementary Material

Supplementary Data S4: STRING network file (.tsv format) containing all high-confidence protein-protein interactions (combined score ≥ 0.700) for the 81-protein PD-CKD convergent network.

#node1	node2	node1_string_id	node2_string_id	neighborhood_on_chromosome	gene_fusion	phylogenetic_cooccurrence	homology	coexpression	experimentally_determined_interaction	database_annotated	automated_textmining	combined_score
ABCB1	CYP2D6	9606.ENSP00000478255	9606.ENSP00000496150	0	0	0	0	0.067	0.069	0	0.766	0.779
ACE	TNF	9606.ENSP00000290866	9606.ENSP00000398698	0	0	0	0	0.044	0	0	0.720	0.720
ACE	IL6	9606.ENSP00000290866	9606.ENSP00000385675	0	0	0	0	0	0	0	0.776	0.776
ACE	INS	9606.ENSP00000290866	9606.ENSP00000380432	0	0	0	0	0.052	0	0	0.847	0.849
ACTN4	WT1	9606.ENSP00000252699	9606.ENSP00000368370	0	0	0	0	0.044	0	0	0.785	0.785
ACTN4	NPHS2	9606.ENSP00000252699	9606.ENSP00000356587	0	0	0	0	0	0	0	0.971	0.971
AKT1	MAPK1	9606.ENSP00000451828	9606.ENSP00000215832	0	0	0	0.593	0.118	0.110	0.900	0.872	0.988
AKT1	IL1B	9606.ENSP00000451828	9606.ENSP00000263341	0	0	0	0	0	0	0	0.864	0.864
AKT1	PPAR	9606.ENSP	9606.ENSP	0	0	0	0	0	0.095	0	0.741	0.756

	GC1A	00000451828	00000264867									
AKT1	HSPA9	9606.ENSP00000451828	9606.ENSP00000297185	0	0	0	0	0.047	0.394	0	0.540	0.711
AKT1	NQO1	9606.ENSP00000451828	9606.ENSP00000319788	0	0	0	0	0.042	0.292	0	0.602	0.706
AKT1	MAPT	9606.ENSP00000451828	9606.ENSP00000340820	0	0	0	0	0	0.737	0	0.534	0.872
AKT1	FN1	9606.ENSP00000451828	9606.ENSP00000346839	0	0	0	0	0.055	0	0	0.844	0.846
AKT1	PARP1	9606.ENSP00000451828	9606.ENSP00000355759	0	0	0	0	0.049	0	0	0.824	0.825
AKT1	NGF	9606.ENSP00000451828	9606.ENSP00000358525	0	0	0	0	0	0	0	0.818	0.818
AKT1	INS	9606.ENSP00000451828	9606.ENSP00000380432	0	0	0	0	0	0.071	0	0.952	0.953
AKT1	IL6	9606.ENSP00000451828	9606.ENSP00000385675	0	0	0	0	0	0	0	0.872	0.872
AKT1	TNF	9606.ENSP00000451828	9606.ENSP00000398698	0	0	0	0	0.074	0.057	0	0.903	0.907
AKT1	BDNF	9606.ENSP00000451828	9606.ENSP00000414303	0	0	0	0	0	0.044	0	0.835	0.835

AKT1	HSPA8	9606.ENSP00000451828	9606.ENSP00000437125	0	0	0	0	0.049	0.521	0	0.697	0.850
AKT1	NOS1	9606.ENSP00000451828	9606.ENSP00000477999	0	0	0	0	0	0.045	0.900	0.554	0.953
APOA1	FN1	9606.ENSP00000236850	9606.ENSP00000346839	0	0	0	0	0.080	0.111	0	0.826	0.845
APOA1	SNCA	9606.ENSP00000236850	9606.ENSP00000500990	0	0	0	0	0.067	0.510	0.400	0.625	0.883
APOA1	INS	9606.ENSP00000236850	9606.ENSP00000380432	0	0	0	0	0.127	0	0.400	0.835	0.906
APOA1	CUBN	9606.ENSP00000236850	9606.ENSP00000367064	0	0	0	0	0	0	0.500	0.972	0.985
ATP13A2	DNAJC13	9606.ENSP00000327214	9606.ENSP00000260818	0	0	0	0	0.055	0	0	0.730	0.734
ATP13A2	FBXO7	9606.ENSP00000327214	9606.ENSP00000266087	0	0	0	0	0.057	0	0	0.883	0.885
ATP13A2	SLC6A3	9606.ENSP00000327214	9606.ENSP00000270349	0	0	0	0	0	0	0	0.816	0.816
ATP13A2	LRRK2	9606.ENSP00000327214	9606.ENSP00000298910	0	0	0	0	0	0.071	0	0.843	0.848
ATP13A2	VPS35	9606.ENSP0000032721	9606.ENSP0000029913	0	0	0	0	0	0.069	0	0.852	0.856

		4	8									
ATP13A2	GBA1	9606.ENSP00000327214	9606.ENSP00000314508	0	0	0	0	0.060	0	0	0.869	0.871
ATP13A2	MAPT	9606.ENSP00000327214	9606.ENSP00000340820	0	0	0	0	0	0.053	0	0.721	0.724
ATP13A2	SYNJ1	9606.ENSP00000327214	9606.ENSP00000409667	0	0	0	0	0.046	0.049	0	0.730	0.734
ATP13A2	VPS13C	9606.ENSP00000327214	9606.ENSP00000493560	0	0	0	0	0.111	0.071	0	0.759	0.783
ATP13A2	DNAJC6	9606.ENSP00000327214	9606.ENSP00000360108	0	0	0	0	0.119	0.071	0	0.763	0.789
ATP13A2	PRKN	9606.ENSP00000327214	9606.ENSP00000355865	0	0	0	0	0	0	0	0.836	0.836
ATP13A2	PLA2G6	9606.ENSP00000327214	9606.ENSP00000333142	0	0	0	0	0.067	0	0	0.840	0.844
ATP13A2	PINK1	9606.ENSP00000327214	9606.ENSP00000364204	0	0	0	0	0.062	0	0	0.922	0.924
ATP13A2	SNCA	9606.ENSP00000327214	9606.ENSP00000500990	0	0	0	0	0.063	0	0	0.940	0.941
ATP13A2	PARK7	9606.ENSP00000327214	9606.ENSP00000340278	0.041	0	0	0	0	0	0	0.957	0.957
BDN	MAP	9606.ENSP	9606.ENSP	0	0	0	0	0	0	0.750	0.407	0.845

F	K1	00000414303	00000215832									
BDNF	GFAP	9606.ENSP00000414303	9606.ENSP00000253408	0	0	0	0	0	0	0	0.804	0.804
BDNF	IL1B	9606.ENSP00000414303	9606.ENSP00000263341	0	0	0	0	0.042	0	0	0.844	0.844
BDNF	MAOA	9606.ENSP00000414303	9606.ENSP00000340684	0	0	0	0	0	0	0	0.741	0.741
BDNF	HTT	9606.ENSP00000414303	9606.ENSP00000347184	0	0	0	0	0	0	0	0.823	0.823
BDNF	DRD2	9606.ENSP00000414303	9606.ENSP00000354859	0	0	0	0	0.057	0	0	0.787	0.790
BDNF	MAOB	9606.ENSP00000414303	9606.ENSP00000367309	0	0	0	0	0	0	0	0.708	0.708
BDNF	TH	9606.ENSP00000414303	9606.ENSP00000370571	0	0	0	0	0	0	0	0.837	0.837
BDNF	DRD1	9606.ENSP00000414303	9606.ENSP00000377353	0	0	0	0	0.067	0	0	0.789	0.795
BDNF	INS	9606.ENSP00000414303	9606.ENSP00000380432	0	0	0	0	0	0	0	0.750	0.750
BDNF	IL6	9606.ENSP00000414303	9606.ENSP00000385675	0	0	0	0	0.050	0	0	0.844	0.846

BDNF	TNF	9606.ENSP00000414303	9606.ENSP00000398698	0	0	0	0	0	0	0	0.739	0.739
BDNF	GDNF	9606.ENSP00000414303	9606.ENSP00000409007	0	0	0	0	0.048	0	0	0.963	0.963
BDNF	SNCA	9606.ENSP00000414303	9606.ENSP00000500990	0	0	0	0	0.045	0	0	0.797	0.798
CFH	CFHR1	9606.ENSP00000356399	9606.ENSP00000314299	0	0	0	0.959	0.123	0.897	0	0.079	0.910
CFHR1	CFHR5	9606.ENSP00000314299	9606.ENSP00000256785	0	0	0	0.941	0.144	0.422	0.500	0.096	0.746
COL4A1	FN1	9606.ENSP00000364979	9606.ENSP00000346839	0	0	0	0	0.250	0.050	0	0.805	0.849
COL4A1	COL4A3	9606.ENSP00000364979	9606.ENSP00000379823	0	0	0.082	0.909	0	0	0.540	0.426	0.736
COL4A3	COL4A5	9606.ENSP00000379823	9606.ENSP00000331902	0	0	0.079	0.919	0	0	0.800	0.518	0.903
COL4A3	COL4A4	9606.ENSP00000379823	9606.ENSP00000379866	0	0	0.115	0.841	0.893	0	0.900	0.535	0.995
COL4A4	COL4A5	9606.ENSP00000379866	9606.ENSP00000331902	0	0	0.097	0.867	0.107	0	0.800	0.469	0.902
COL4A4	NPHS2	9606.ENSP0000037986	9606.ENSP0000035658	0	0	0	0	0.044	0	0	0.762	0.763

		6	7									
CYP1A1	GSTM1	9606.ENSP00000378488	9606.ENSP00000311469	0	0	0	0	0.067	0.045	0.900	0.918	0.991
CYP1A1	NQO1	9606.ENSP00000378488	9606.ENSP00000319788	0	0	0	0	0.124	0	0	0.722	0.746
CYP1A1	CYP2D6	9606.ENSP00000378488	9606.ENSP00000496150	0	0	0.138	0.802	0.057	0	0.911	0.220	0.936
CYP1A1	GSTP1	9606.ENSP00000378488	9606.ENSP00000381607	0	0	0	0	0.109	0.045	0.900	0.735	0.974
CYP2D6	NAT2	9606.ENSP00000496150	9606.ENSP00000286479	0	0	0	0	0.053	0	0	0.783	0.786
CYP2D6	MAOA	9606.ENSP00000496150	9606.ENSP00000340684	0	0	0	0	0.062	0	0.900	0.641	0.963
CYP2D6	MAOB	9606.ENSP00000496150	9606.ENSP00000367309	0	0	0	0	0.067	0	0.900	0.620	0.961
CYP2D6	GSTP1	9606.ENSP00000496150	9606.ENSP00000381607	0	0	0	0	0.109	0.045	0	0.713	0.734
DBH	SLC6A3	9606.ENSP00000376776	9606.ENSP00000270349	0	0	0	0	0.058	0	0	0.812	0.815
DBH	MAOA	9606.ENSP00000376776	9606.ENSP00000340684	0	0	0	0	0.054	0	0.900	0.876	0.987
DBH	DRD	9606.ENSP	9606.ENSP	0	0	0	0	0.116	0	0	0.726	0.748

	2	0000037677 6	0000035485 9									
DBH	MAO B	9606.ENSP 0000037677 6	9606.ENSP 0000036730 9	0	0	0	0	0.054	0	0.900	0.849	0.984
DBH	TH	9606.ENSP 0000037677 6	9606.ENSP 0000037057 1	0	0	0	0	0.152	0	0	0.970	0.973
DBH	SLC1 8A2	9606.ENSP 0000037677 6	9606.ENSP 0000049633 9	0	0	0	0	0.047	0	0	0.700	0.702
DBH	DDC	9606.ENSP 0000037677 6	9606.ENSP 0000040364 4	0	0	0	0	0.083	0	0.914	0.819	0.984
DDC	SLC6 A3	9606.ENSP 0000040364 4	9606.ENSP 0000027034 9	0.042	0	0	0	0.055	0	0	0.860	0.862
DDC	MAO A	9606.ENSP 0000040364 4	9606.ENSP 0000034068 4	0	0	0	0	0.063	0.067	0.900	0.863	0.986
DDC	MAO B	9606.ENSP 0000040364 4	9606.ENSP 0000036730 9	0	0	0	0	0.048	0.067	0.900	0.872	0.987
DDC	TH	9606.ENSP 0000040364 4	9606.ENSP 0000037057 1	0	0	0	0	0.079	0.071	0.914	0.943	0.995
DDC	SLC1 8A2	9606.ENSP 0000040364 4	9606.ENSP 0000049633 9	0	0	0	0	0	0.095	0	0.965	0.967
DNA JC13	VPS1 3C	9606.ENSP 0000026081 8	9606.ENSP 0000049356 0	0	0	0	0	0.125	0	0	0.691	0.718

DNAJC13	TMEM230	9606.ENSP00000260818	9606.ENSP00000341364	0	0	0	0	0.044	0	0	0.730	0.731
DNAJC13	SYNJ1	9606.ENSP00000260818	9606.ENSP00000409667	0	0	0	0	0.093	0	0	0.727	0.742
DNAJC13	DNAJC6	9606.ENSP00000260818	9606.ENSP00000360108	0	0	0	0	0.111	0	0	0.810	0.824
DNAJC13	HSPA8	9606.ENSP00000260818	9606.ENSP00000437125	0	0	0	0	0.044	0.453	0	0.716	0.838
DNAJC13	VPS35	9606.ENSP00000260818	9606.ENSP00000299138	0	0	0	0	0.151	0.292	0	0.904	0.937
DNAJC6	FBXO7	9606.ENSP00000360108	9606.ENSP00000266087	0	0	0	0	0.053	0	0	0.760	0.763
DNAJC6	VPS35	9606.ENSP00000360108	9606.ENSP00000299138	0	0	0	0	0.119	0.067	0	0.796	0.817
DNAJC6	PLA2G6	9606.ENSP00000360108	9606.ENSP00000333142	0	0	0	0	0.058	0	0	0.738	0.743
DNAJC6	PRKN	9606.ENSP00000360108	9606.ENSP00000355865	0	0	0	0	0.050	0.055	0	0.718	0.725
DNAJC6	PINK1	9606.ENSP00000360108	9606.ENSP00000364204	0	0	0	0	0.063	0	0	0.719	0.725
DNAJC6	VPS13C	9606.ENSP0000036010	9606.ENSP0000049356	0	0	0	0	0	0.095	0	0.747	0.762

		8	0									
DNAJC6	SYNJ1	9606.ENSP00000360108	9606.ENSP00000409667	0	0	0	0	0.086	0.070	0	0.906	0.913
DNAJC6	HSPA8	9606.ENSP00000360108	9606.ENSP00000437125	0	0	0	0	0	0.904	0.900	0.996	0.999
DRD1	SLC6A3	9606.ENSP00000377353	9606.ENSP00000270349	0	0	0	0	0	0	0	0.906	0.906
DRD2	SLC6A3	9606.ENSP00000354859	9606.ENSP00000270349	0	0	0	0	0.054	0.380	0.900	0.995	0.999
DRD2	MAOA	9606.ENSP00000354859	9606.ENSP00000340684	0	0	0	0	0	0	0	0.736	0.736
DRD2	SLC18A2	9606.ENSP00000354859	9606.ENSP00000496339	0	0	0	0	0.042	0	0	0.814	0.814
DRD2	TH	9606.ENSP00000354859	9606.ENSP00000370571	0	0	0	0	0.085	0	0	0.834	0.841
FBXO7	HTRA2	9606.ENSP00000266087	9606.ENSP00000258080	0	0	0	0	0.064	0	0	0.773	0.778
FBXO7	UCHL1	9606.ENSP00000266087	9606.ENSP00000284440	0	0	0	0	0.047	0	0	0.776	0.777
FBXO7	GBA1	9606.ENSP00000266087	9606.ENSP00000314508	0	0	0	0	0.049	0	0	0.784	0.786
FBXO	SNC	9606.ENSP	9606.ENSP	0	0	0	0	0.391	0	0	0.669	0.789

7	A	00000266087	00000500990									
FBXO7	LRRK2	9606.ENSP00000266087	9606.ENSP00000298910	0	0	0	0	0.047	0.091	0	0.808	0.819
FBXO7	VPS35	9606.ENSP00000266087	9606.ENSP00000299138	0	0	0	0	0.049	0	0	0.843	0.845
FBXO7	PARK7	9606.ENSP00000266087	9606.ENSP00000340278	0	0	0	0	0.049	0	0	0.863	0.864
FBXO7	PLA2G6	9606.ENSP00000266087	9606.ENSP00000333142	0	0	0	0	0.045	0	0	0.897	0.897
FBXO7	PRKN	9606.ENSP00000266087	9606.ENSP00000355865	0	0	0	0	0	0.331	0	0.882	0.917
FBXO7	PINK1	9606.ENSP00000266087	9606.ENSP00000364204	0	0	0	0	0.149	0.510	0	0.989	0.995
FN1	MAPK1	9606.ENSP00000346839	9606.ENSP00000215832	0	0	0	0	0	0	0.500	0.455	0.716
FN1	IL1B	9606.ENSP00000346839	9606.ENSP00000263341	0	0	0	0	0.055	0.051	0	0.866	0.869
FN1	SOD1	9606.ENSP00000346839	9606.ENSP00000270142	0	0	0	0	0.053	0	0	0.771	0.773
FN1	INS	9606.ENSP00000346839	9606.ENSP00000380432	0	0	0	0	0.055	0	0	0.697	0.701

FN1	IL6	9606.ENSP00000346839	9606.ENSP00000385675	0	0	0	0	0.087	0	0	0.895	0.900
FN1	TNF	9606.ENSP00000346839	9606.ENSP00000398698	0	0	0	0	0.057	0	0	0.970	0.970
GBA1	UCHL1	9606.ENSP00000314508	9606.ENSP00000284440	0	0	0	0	0.103	0	0	0.690	0.710
GBA1	LRRK2	9606.ENSP00000314508	9606.ENSP00000298910	0	0	0	0	0.051	0	0	0.922	0.922
GBA1	VPS35	9606.ENSP00000314508	9606.ENSP00000299138	0	0	0	0	0.049	0	0	0.755	0.757
GBA1	PARK7	9606.ENSP00000314508	9606.ENSP00000340278	0	0	0	0	0.042	0	0	0.744	0.745
GBA1	PLA2G6	9606.ENSP00000314508	9606.ENSP00000333142	0	0	0	0	0	0	0	0.751	0.751
GBA1	PINK1	9606.ENSP00000314508	9606.ENSP00000364204	0	0	0	0	0.056	0	0	0.756	0.760
GBA1	PRKN	9606.ENSP00000314508	9606.ENSP00000355865	0	0	0	0	0	0.292	0	0.879	0.911
GBA1	SNCA	9606.ENSP00000314508	9606.ENSP00000500990	0	0	0	0	0	0.512	0	0.928	0.963
GDNF	NGF	9606.ENSP0000040900	9606.ENSP0000035852	0	0	0	0	0.060	0	0	0.925	0.926

		7	5									
GDNF	TH	9606.ENSP00000409007	9606.ENSP00000370571	0	0	0	0	0.045	0	0	0.847	0.847
GFAP	TNF	9606.ENSP00000253408	9606.ENSP00000398698	0	0	0	0	0.086	0.091	0	0.674	0.705
GFAP	SOD1	9606.ENSP00000253408	9606.ENSP00000270142	0	0	0	0	0	0	0	0.718	0.718
GFAP	MAPT	9606.ENSP00000253408	9606.ENSP00000340820	0	0	0	0	0.216	0.187	0	0.630	0.743
GFAP	IL6	9606.ENSP00000253408	9606.ENSP00000385675	0	0	0	0	0.080	0	0	0.772	0.782
GFAP	TH	9606.ENSP00000253408	9606.ENSP00000370571	0	0	0	0	0.087	0	0	0.775	0.786
GFAP	HSPA8	9606.ENSP00000253408	9606.ENSP00000437125	0	0	0	0	0.059	0.129	0.700	0.299	0.804
GFAP	IL1B	9606.ENSP00000253408	9606.ENSP00000263341	0	0	0	0	0.067	0	0	0.815	0.820
GSTM1	NAT2	9606.ENSP00000311469	9606.ENSP00000286479	0	0	0	0	0.042	0.053	0	0.772	0.775
GSTM1	NQO1	9606.ENSP00000311469	9606.ENSP00000319788	0	0	0	0	0.042	0	0	0.719	0.719
GST	GSTP	9606.ENSP	9606.ENSP	0	0	0	0.77	0.069	0.112	0.700	0.317	0.808

M1	1	0000031146<br>9	0000038160<br>7				4					
GSTP<br>1	SOD1	9606.ENSP<br>0000038160<br>7	9606.ENSP<br>0000027014<br>2	0	0	0	0	0.107	0.164	0	0.652	0.718
GSTP<br>1	NQO<br>1	9606.ENSP<br>0000038160<br>7	9606.ENSP<br>0000031978<br>8	0	0	0	0	0.087	0	0	0.714	0.728
HNM<br>T	MAO<br>B	9606.ENSP<br>0000028009<br>7	9606.ENSP<br>0000036730<br>9	0	0	0	0	0.126	0	0.900	0.447	0.947
HNM<br>T	MAO<br>A	9606.ENSP<br>0000028009<br>7	9606.ENSP<br>0000034068<br>4	0	0	0	0	0.129	0	0.900	0.452	0.948
HSP<br>A8	TAR<br>DBP	9606.ENSP<br>0000043712<br>5	9606.ENSP<br>0000024018<br>5	0	0	0	0	0.266	0.175	0	0.632	0.758
HSP<br>A8	SOD1	9606.ENSP<br>0000043712<br>5	9606.ENSP<br>0000027014<br>2	0	0	0	0	0.249	0.406	0	0.733	0.870
HSP<br>A8	UCH<br>L1	9606.ENSP<br>0000043712<br>5	9606.ENSP<br>0000028444<br>0	0	0	0	0	0.072	0.510	0	0.848	0.924
HSP<br>A8	HSP<br>A9	9606.ENSP<br>0000043712<br>5	9606.ENSP<br>0000029718<br>5	0	0	0.076	0.92<br>9	0.260	0.644	0.500	0.634	0.947
HSP<br>A8	LRR<br>K2	9606.ENSP<br>0000043712<br>5	9606.ENSP<br>0000029891<br>0	0	0	0	0	0	0.855	0	0.581	0.936
HSP<br>A8	PAR<br>K7	9606.ENSP<br>0000043712<br>5	9606.ENSP<br>0000034027<br>8	0.057	0	0	0	0.158	0.125	0.500	0.500	0.794

HSPA8	MAPT	9606.ENSP00000437125	9606.ENSP00000340820	0	0	0	0	0	0.872	0	0.615	0.948
HSPA8	HTT	9606.ENSP00000437125	9606.ENSP00000347184	0	0	0	0	0.067	0.568	0	0.820	0.921
HSPA8	PRKN	9606.ENSP00000437125	9606.ENSP00000355865	0	0	0	0	0	0.630	0	0.743	0.901
HSPA8	SNCA	9606.ENSP00000437125	9606.ENSP00000500990	0	0	0	0	0.068	0.670	0	0.962	0.987
HSPA9	SNCA	9606.ENSP00000297185	9606.ENSP00000500990	0	0	0	0	0.059	0.180	0	0.666	0.720
HSPA9	PINK1	9606.ENSP00000297185	9606.ENSP00000364204	0	0	0	0	0	0.079	0	0.714	0.725
HSPA9	PRKN	9606.ENSP00000297185	9606.ENSP00000355865	0	0	0	0	0	0.484	0	0.642	0.807
HSPA9	PARK7	9606.ENSP00000297185	9606.ENSP00000340278	0.057	0	0	0	0.119	0.354	0	0.988	0.992
HTRA2	SNCA	9606.ENSP00000258080	9606.ENSP00000500990	0	0	0	0	0.043	0	0	0.719	0.719
HTRA2	UCHL1	9606.ENSP00000258080	9606.ENSP00000284440	0	0	0	0	0.047	0	0	0.721	0.722
HTRA2	PARK7	9606.ENSP0000025808	9606.ENSP0000034027	0	0	0	0	0.074	0	0	0.713	0.722

		0	8									
HTRA2	PLA2G6	9606.ENSP00000258080	9606.ENSP00000333142	0	0	0	0	0.049	0	0	0.751	0.753
HTRA2	TNF	9606.ENSP00000258080	9606.ENSP00000398698	0	0	0	0	0.057	0.743	0	0.325	0.822
HTRA2	PRKN	9606.ENSP00000258080	9606.ENSP00000355865	0	0	0	0	0	0.314	0	0.819	0.871
HTRA2	PINK1	9606.ENSP00000258080	9606.ENSP00000364204	0	0	0	0	0.074	0.358	0.900	0.914	0.994
HTT	TARDBP	9606.ENSP00000347184	9606.ENSP00000240185	0	0	0	0	0.056	0.094	0	0.897	0.905
HTT	PPARGC1A	9606.ENSP00000347184	9606.ENSP00000264867	0	0	0	0	0.055	0	0.900	0.824	0.982
HTT	SOD1	9606.ENSP00000347184	9606.ENSP00000270142	0	0	0	0	0.055	0.090	0	0.844	0.855
HTT	LRRK2	9606.ENSP00000347184	9606.ENSP00000298910	0	0	0	0	0.072	0.301	0	0.580	0.704
HTT	MAPT	9606.ENSP00000347184	9606.ENSP00000340820	0	0	0	0	0.042	0.095	0	0.698	0.715
HTT	PRKN	9606.ENSP00000347184	9606.ENSP00000355865	0	0	0	0	0	0	0	0.817	0.817
HTT	SNC	9606.ENSP	9606.ENSP	0	0	0	0	0.057	0.422	0	0.982	0.989

	A	00000347184	00000500990									
IGF2	WT1	9606.ENSP00000391826	9606.ENSP00000368370	0	0	0	0	0.044	0	0	0.812	0.812
IGF2	INS	9606.ENSP00000391826	9606.ENSP00000380432	0	0	0	0	0.044	0	0	0.981	0.981
IL1B	NGF	9606.ENSP00000263341	9606.ENSP00000358525	0	0	0	0	0.042	0	0	0.728	0.728
IL1B	SOD2	9606.ENSP00000263341	9606.ENSP00000446252	0	0	0	0	0.305	0	0	0.733	0.806
IL1B	UMOD	9606.ENSP00000263341	9606.ENSP00000379438	0	0	0	0	0.057	0	0	0.848	0.850
IL1B	INS	9606.ENSP00000263341	9606.ENSP00000380432	0	0	0	0	0.068	0	0	0.893	0.896
IL1B	IL6	9606.ENSP00000263341	9606.ENSP00000385675	0	0	0	0	0.515	0	0.400	0.989	0.996
IL1B	TNF	9606.ENSP00000263341	9606.ENSP00000398698	0	0	0	0	0.616	0	0.400	0.993	0.998
IL6	MAPK1	9606.ENSP00000385675	9606.ENSP00000215832	0	0	0	0	0.055	0	0.400	0.897	0.936
IL6	NGF	9606.ENSP00000385675	9606.ENSP00000358525	0	0	0	0	0.086	0	0	0.734	0.747

IL6	INS	9606.ENSP00000385675	9606.ENSP00000380432	0	0	0	0	0	0	0	0.933	0.933
IL6	TNF	9606.ENSP00000385675	9606.ENSP00000398698	0	0	0	0	0.261	0	0.400	0.989	0.994
INS	MAPK1	9606.ENSP00000380432	9606.ENSP00000215832	0	0	0	0	0.055	0.096	0	0.745	0.763
INS	PPARGC1A	9606.ENSP00000380432	9606.ENSP00000264867	0	0	0	0	0	0	0.400	0.888	0.930
INS	NGF	9606.ENSP00000380432	9606.ENSP00000358525	0	0	0	0	0.098	0	0	0.689	0.707
INS	TH	9606.ENSP00000380432	9606.ENSP00000370571	0	0.098	0	0	0.060	0	0	0.702	0.725
INS	SNCA	9606.ENSP00000380432	9606.ENSP00000500990	0	0	0	0	0	0	0.400	0.835	0.896
INS	TNF	9606.ENSP00000380432	9606.ENSP00000398698	0	0	0	0	0	0	0	0.926	0.926
KLK6	SNCA	9606.ENSP00000366047	9606.ENSP00000500990	0	0	0	0	0	0.312	0	0.604	0.716
LRRK2	TARDBP	9606.ENSP00000298910	9606.ENSP00000240185	0	0	0	0	0.087	0.314	0	0.698	0.794
LRRK2	SLC6A3	9606.ENSP0000029891	9606.ENSP0000027034	0	0	0	0	0	0	0	0.749	0.749

		0	9									
LRRK2	UCHL1	9606.ENSP00000298910	9606.ENSP00000284440	0	0	0	0	0	0.306	0	0.784	0.844
LRRK2	SYNJ1	9606.ENSP00000298910	9606.ENSP00000409667	0	0	0	0	0.068	0.510	0	0.599	0.800
LRRK2	VPS35	9606.ENSP00000298910	9606.ENSP00000299138	0	0	0	0	0	0.331	0	0.862	0.904
LRRK2	MAPT	9606.ENSP00000298910	9606.ENSP00000340820	0	0	0	0	0	0.628	0	0.826	0.932
LRRK2	PINK1	9606.ENSP00000298910	9606.ENSP00000364204	0	0	0	0	0.048	0.046	0	0.954	0.955
LRRK2	SNCA	9606.ENSP00000298910	9606.ENSP00000500990	0	0	0	0	0.071	0.512	0	0.973	0.986
LRRK2	PRKN	9606.ENSP00000298910	9606.ENSP00000355865	0	0	0	0	0.066	0.299	0	0.981	0.986
LRRK2	PARK7	9606.ENSP00000298910	9606.ENSP00000340278	0	0	0	0	0	0.294	0	0.984	0.988
MAOA	SLC6A3	9606.ENSP00000340684	9606.ENSP00000270349	0	0	0	0	0	0	0	0.890	0.890
MAOA	SNCA	9606.ENSP00000340684	9606.ENSP00000500990	0	0	0	0	0.057	0	0	0.706	0.710
MAO	TH	9606.ENSP	9606.ENSP	0	0	0	0	0	0	0	0.785	0.785

A		00000340684	00000370571									
MAOA	SLC18A2	9606.ENSP00000340684	9606.ENSP00000496339	0	0	0	0	0.042	0	0	0.792	0.793
MAOA	PRKN	9606.ENSP00000340684	9606.ENSP00000355865	0	0	0	0	0	0.097	0.900	0.489	0.949
MAOA	MAOB	9606.ENSP00000340684	9606.ENSP00000367309	0	0	0.052	0.976	0.163	0.609	0.900	0.063	0.965
MAOB	SLC6A3	9606.ENSP00000367309	9606.ENSP00000270349	0	0	0	0	0	0	0	0.882	0.882
MAOB	PRKN	9606.ENSP00000367309	9606.ENSP00000355865	0	0	0	0	0.049	0	0.900	0.716	0.970
MAOB	PINK1	9606.ENSP00000367309	9606.ENSP00000364204	0	0	0	0	0.120	0	0	0.720	0.743
MAOB	SLC18A2	9606.ENSP00000367309	9606.ENSP00000496339	0	0	0	0	0.042	0	0	0.791	0.792
MAOB	TH	9606.ENSP00000367309	9606.ENSP00000370571	0	0	0	0	0	0	0	0.792	0.792
MAOB	SNCA	9606.ENSP00000367309	9606.ENSP00000500990	0	0	0	0	0.042	0	0	0.815	0.815
MAPK1	TNF	9606.ENSP00000215832	9606.ENSP00000398698	0	0	0	0	0.083	0	0	0.746	0.757

MAPK1	NOS1	9606.ENSP00000215832	9606.ENSP00000477999	0	0	0	0	0.067	0	0.900	0.167	0.915
MAPK1	MAPT	9606.ENSP00000215832	9606.ENSP00000340820	0	0	0	0	0.055	0.314	0.900	0.348	0.952
MAPT	TARDBP	9606.ENSP00000340820	9606.ENSP00000240185	0	0	0	0	0	0.049	0	0.970	0.970
MAPT	UCHL1	9606.ENSP00000340820	9606.ENSP00000284440	0	0	0	0	0.087	0.095	0	0.690	0.721
MAPT	PARK7	9606.ENSP00000340820	9606.ENSP00000340278	0	0	0	0	0	0	0	0.772	0.772
MAPT	SNCB	9606.ENSP00000340820	9606.ENSP00000377296	0	0	0	0	0.190	0	0	0.644	0.700
MAPT	PINK1	9606.ENSP00000340820	9606.ENSP00000364204	0	0	0	0	0.060	0	0	0.751	0.756
MAPT	PRKN	9606.ENSP00000340820	9606.ENSP00000355865	0	0	0	0	0.074	0.623	0	0.789	0.920
MAPT	SNCA	9606.ENSP00000340820	9606.ENSP00000500990	0	0	0	0	0.079	0.817	0	0.970	0.994
NGF	TNF	9606.ENSP00000358525	9606.ENSP00000398698	0	0	0	0	0	0	0	0.714	0.714
NGF	TH	9606.ENSP0000035852	9606.ENSP0000037057	0	0	0	0	0	0	0	0.791	0.791

		5	1									
NPHS2	WT1	9606.ENSP00000356587	9606.ENSP00000368370	0	0	0	0	0.056	0.064	0	0.917	0.921
NQO1	TNF	9606.ENSP00000319788	9606.ENSP00000398698	0	0	0	0	0	0.324	0	0.592	0.712
NR4A2	SLC6A3	9606.ENSP00000344479	9606.ENSP00000270349	0	0	0	0	0	0	0	0.788	0.788
NR4A2	SNCA	9606.ENSP00000344479	9606.ENSP00000500990	0	0	0	0	0	0	0	0.748	0.748
NR4A2	SLC18A2	9606.ENSP00000344479	9606.ENSP00000496339	0	0	0	0	0	0	0	0.802	0.802
NR4A2	TH	9606.ENSP00000344479	9606.ENSP00000370571	0	0	0	0	0	0	0	0.837	0.837
PARK7	SOD1	9606.ENSP00000340278	9606.ENSP00000270142	0.055	0	0	0	0.815	0.643	0.792	0.819	0.997
PARK7	UCHL1	9606.ENSP00000340278	9606.ENSP00000284440	0	0	0	0	0.060	0.162	0	0.822	0.848
PARK7	VPS35	9606.ENSP00000340278	9606.ENSP00000299138	0	0	0	0	0.109	0	0	0.831	0.843
PARK7	PLA2G6	9606.ENSP00000340278	9606.ENSP00000333142	0.061	0	0	0	0	0	0	0.811	0.815
PAR	SOD2	9606.ENSP	9606.ENSP	0	0	0	0	0.140	0	0.792	0.605	0.923

K7		00000340278	00000446252									
PARK7	SNCA	9606.ENSP00000340278	9606.ENSP00000500990	0	0	0	0	0	0.631	0	0.985	0.994
PARK7	PINK1	9606.ENSP00000340278	9606.ENSP00000364204	0	0	0	0	0	0.527	0	0.999	0.999
PARK7	PRKN	9606.ENSP00000340278	9606.ENSP00000355865	0	0	0	0	0	0.735	0.500	0.996	0.999
PARP1	TNF	9606.ENSP00000355759	9606.ENSP00000398698	0	0	0	0	0	0	0	0.735	0.735
PARP1	SOD2	9606.ENSP00000355759	9606.ENSP00000446252	0	0	0	0	0.042	0	0	0.774	0.774
PINK1	PPARGC1A	9606.ENSP00000364204	9606.ENSP00000264867	0	0	0	0	0.042	0	0	0.759	0.760
PINK1	UCHL1	9606.ENSP00000364204	9606.ENSP00000284440	0	0	0	0	0.042	0	0	0.788	0.788
PINK1	VPS35	9606.ENSP00000364204	9606.ENSP00000299138	0	0	0	0	0.042	0.088	0	0.846	0.854
PINK1	PLA2G6	9606.ENSP00000364204	9606.ENSP00000333142	0	0	0	0	0	0	0	0.800	0.800
PINK1	PRKN	9606.ENSP00000364204	9606.ENSP00000355865	0	0	0	0	0	0.979	0.900	0.999	0.999

PINK1	TFAM	9606.ENSP00000364204	9606.ENSP00000420588	0	0	0	0	0	0	0	0.810	0.810
PINK1	SNCA	9606.ENSP00000364204	9606.ENSP00000500990	0	0	0	0	0.128	0.518	0	0.947	0.975
PKD1	PKD2	9606.ENSP00000262304	9606.ENSP00000237596	0	0	0	0.570	0.045	0.987	0.540	0.993	0.999
PLA2G6	UCHL1	9606.ENSP00000333142	9606.ENSP00000284440	0	0	0	0	0	0	0	0.704	0.704
PLA2G6	VPS35	9606.ENSP00000333142	9606.ENSP00000299138	0	0	0	0	0.074	0.095	0	0.750	0.773
PLA2G6	VPS13C	9606.ENSP00000333142	9606.ENSP00000493560	0	0	0	0	0.074	0	0	0.700	0.710
PLA2G6	SYNJ1	9606.ENSP00000333142	9606.ENSP00000409667	0	0	0	0	0.053	0	0	0.721	0.724
POLG	TFAM	9606.ENSP00000399851	9606.ENSP00000420588	0	0	0	0	0.049	0.329	0	0.929	0.951
PPARGC1A	SOD1	9606.ENSP00000264867	9606.ENSP00000270142	0	0	0	0	0	0	0.900	0.598	0.958
PPARGC1A	SOD2	9606.ENSP00000264867	9606.ENSP00000446252	0	0	0	0	0	0	0.900	0.741	0.973
PPARGC1	TFAM	9606.ENSP0000026486	9606.ENSP0000042058	0	0	0	0	0.042	0	0.900	0.893	0.988

A		7	8									
PRKN	TARDBP	9606.ENSP00000355865	9606.ENSP00000240185	0	0	0	0	0	0.601	0.900	0.644	0.984
PRKN	SLC6A3	9606.ENSP00000355865	9606.ENSP00000270349	0	0	0	0	0	0.305	0	0.778	0.839
PRKN	UCHL1	9606.ENSP00000355865	9606.ENSP00000284440	0	0	0	0	0	0.563	0	0.913	0.960
PRKN	VPS35	9606.ENSP00000355865	9606.ENSP00000299138	0	0	0	0	0	0.359	0	0.898	0.932
PRKN	TH	9606.ENSP00000355865	9606.ENSP00000370571	0	0	0	0	0	0	0	0.764	0.764
PRKN	TFAM	9606.ENSP00000355865	9606.ENSP00000420588	0	0	0	0	0	0	0	0.823	0.823
PRKN	SNCA	9606.ENSP00000355865	9606.ENSP00000500990	0	0	0	0	0	0.832	0.900	0.999	0.999
SLC18A2	SLC6A3	9606.ENSP00000496339	9606.ENSP00000270349	0	0	0	0	0.140	0.296	0	0.949	0.966
SLC18A2	TH	9606.ENSP00000496339	9606.ENSP00000370571	0	0	0	0	0	0.095	0	0.964	0.966
SLC18A2	SNCA	9606.ENSP00000496339	9606.ENSP00000500990	0	0	0	0	0.044	0	0.900	0.805	0.979
SLC6	TH	9606.ENSP	9606.ENSP	0	0	0	0	0.147	0	0	0.944	0.950

A3		00000270349	00000370571									
SLC6A3	SNCA	9606.ENSP00000270349	9606.ENSP00000500990	0	0	0	0	0.058	0.829	0	0.980	0.996
SNCA	TARDBP	9606.ENSP00000500990	9606.ENSP00000240185	0	0	0	0	0	0.063	0	0.994	0.994
SNCA	SOD1	9606.ENSP00000500990	9606.ENSP00000270142	0	0	0	0	0.053	0.292	0	0.859	0.897
SNCA	UCHL1	9606.ENSP00000500990	9606.ENSP00000284440	0	0	0	0	0.089	0.292	0	0.940	0.957
SNCA	VPS35	9606.ENSP00000500990	9606.ENSP00000299138	0	0	0	0	0	0	0	0.778	0.778
SNCA	TH	9606.ENSP00000500990	9606.ENSP00000370571	0	0	0	0	0	0.095	0.900	0.975	0.997
SNCA	SNCB	9606.ENSP00000500990	9606.ENSP00000377296	0	0	0	0.947	0.146	0.577	0	0.562	0.828
SNCB	UCHL1	9606.ENSP00000377296	9606.ENSP00000284440	0	0	0	0	0.191	0	0	0.667	0.719
SOD1	TARDBP	9606.ENSP00000270142	9606.ENSP00000240185	0	0	0	0	0.077	0	0	0.989	0.989
SOD1	SOD2	9606.ENSP00000270142	9606.ENSP00000446252	0	0	0	0	0.201	0.661	0.900	0.821	0.994

SOD2	TFAM	9606.ENSP00000446252	9606.ENSP00000420588	0	0	0	0	0.084	0	0	0.795	0.805
SYNJ1	VPS35	9606.ENSP00000409667	9606.ENSP00000299138	0	0	0	0	0	0.063	0	0.740	0.746
SYNJ1	VPS13C	9606.ENSP00000409667	9606.ENSP00000493560	0	0	0	0	0.043	0.131	0	0.671	0.702
TNF	UMOD	9606.ENSP00000398698	9606.ENSP00000379438	0	0	0	0	0.060	0.071	0	0.691	0.707
VPS13C	VPS35	9606.ENSP00000493560	9606.ENSP00000299138	0	0	0	0	0.118	0.125	0	0.751	0.791
